# Palmatine ameliorates high fat diet induced impaired glucose tolerance

**DOI:** 10.1186/s40659-020-00308-0

**Published:** 2020-09-14

**Authors:** Xusheng Tian, Yukun Zhang, Han Li, Yunfeng Li, Ning Wang, Wei Zhang, Boyan Ma

**Affiliations:** 1 Teaching and Research Department of Theories of Schools of Traditional Chinese Medicine, School of Basic Medical Sciences, Heilongjiang University of Chinese Medicine, Harbin, Heilongjiang 150040 People’s Republic of China; 2 Laboratory of Anatomy, Experimental and Training Center, Heilongjiang University of Chinese Medicine, Harbin, Heilongjiang 150040 People’s Republic of China; 3 Department of Febrile Disease, School of Basic Medical Sciences, Heilongjiang University of Chinese Medicine, 24 Heping Road, Harbin, Heilongjiang 150040 People’s Republic of China; 4 Department of Chinese Medicinal Formulae, School of Basic Medical Sciences, Heilongjiang University of Chinese Medicine, Harbin, Heilongjiang 150040 People’s Republic of China

**Keywords:** Palmatine, Impaired glucose tolerance, Insulin resistance, ERK signaling

## Abstract

**Background:**

The impaired glucose tolerance (IGT) is a representative prediabetes characterized by defective glucose homeostasis, and palmatine (PAL) is a natural isoquinoline alkaloid with multiple pharmacological effects. Our study aims to investigate the therapeutic effect of PAL on the impaired glucose tolerance.

**Methods:**

Male Sprague–Dawley rats were used to establish an IGT model with high fat diet (HFD). Oral glucose tolerance test (OGTT) and further biochemical analysis were conducted to determine the effect of PAL on glucose intolerance in vivo. Molecular details were clarified in a cellular model of IGT induced by Palmitate (PA) on INS-1 cells.

**Results:**

Our study demonstrated a relief of IGT with improved insulin resistance in HFD induced rats after PAL treatment. Besides, promoted pancreas islets function was validated with significantly increased β cell mass after the treatment of PAL. We further found out that PAL could alleviate the β cell apoptosis that accounts for β cell mass loss in IGT model. Moreover, MAPK signaling was investigated in vivo and vitro with the discovery that PAL regulated the MAPK signaling by restricting the ERK and JNK cascades. The insulin secretion assay indicated that PAL significantly promoted the defective insulin secretion in PA-induced INS-1 cells via JNK rather than ERK signaling. Furthermore, PAL treatment was determined to significantly suppress β cell apoptosis in PA-induced cells. We thus thought that PAL promoted the PA-induced impaired insulin release by inhibiting the β cell apoptosis and JNK signaling in vitro.

**Conclusion:**

In summary, PAL ameliorates HFD-induced IGT with novel mechanisms.

## Background

Diabetes mellitus (DM) is a metabolic disease with progressive proceeding and characterized by hyperglycemia [1]. Type 2 diabetes (T2D) is the most typical form of diabetes with an increasing prevalence all over the world [1, 2]. It is documented that T2D accounts for the deaths of about 1.5 million people each year and results in great economic burden for the global healthcare [2]. To avoid the health implication and financial burden caused by T2D, primary prevention is fully necessary. The impaired glucose tolerance (IGT) is considered as a representative prediabetes and emerges before the onset of T2D [3, 4]. Individuals diagnosed with IGT are reported to show high potential to develop T2D [5]. The period of IGT has been regarded as the therapeutic target for the possible treatment of T2D, and appropriate intervention on IGT individuals is believed to slow down or prevent the development of T2D. IGT can be induced by the unhealthy lifestyles such as high fat diet (HFD), smoking, alcohol intake and physical inactivity [6, 7]. In general, approximately 20%–70% of high risk population with prediabetes display obesity, unhealthy eating habits and exercise deficiency [7]. Thus, developing a good lifestyle could help to improve the IGT and reduce the risk of T2D [8]. Moreover, several pharmacological treatments of glucose intolerance have also been demonstrated while with unavoidable side effects. Thus, more efforts should be made to investigate the potential therapeutic agents of IGT.

Palmatine (PAL) is a kind of isoquinoline alkaloids which is widely distributed among traditional Chinese medicines (TCMs) including Coptis chinensis Franch, and tinospora cordifolia [9]. Reportedly, these TCMs have shown anti-diabetic effects on patients with DM [10]. Besides, PAL has been used as a compensatory treatment for celiodynia and gastroenteritis without significant side effects or toxicity [11, 12]. The hepatotoxicity induced by gentamicin could be reduced in PAL-treated rats [13]. Actually, PAL has been reported to possess multiple pharmacological effects including the anti-tumor, anti-bacteria, anti-viral, anti-oxidative, and anti-inflammatory properties together with blood lipid regulation activities [9, 14, 15]. Specially, PAL significantly inhibits the activation of TRIF-dependent NF-κB pathway to alleviate the inflammatory response [16]. The tumor cell proliferation and invasion in prostate cancer is also highly restricted after the treatment of PAL [17]. However, the effect of PAL on prediabetes remains unknown. Recent studies only show that PAL could facilitate the insulin secretion in RINm5F cells [14] and display anti-diabetic effect by meditating the expression of Glu-4 in L6 myotubes [18]. It is further reported that diabetic neuropathic pain and depression are linked with PAL in rats [19]. We thus assume that PAL might have the potential to prevent and treat the prediabetes.

In our study, we aim to figure out whether PAL is implicated in the regulation of IGT and highlights novel mechanisms for the therapeutic effect of PAL on IGT treatment.

## Materials and methods

### Reagents and antibodies

Palmatine for experiment in vivo (purity > 98%, P110208) and JNK inhibitor (SP600125, S125267) were purchased from Aladdin regents. Palmatine for experiment in vitro (purity > 98%, HY-N0110) and ERK inhibitor (PD98059, HY-12028) were purchased from MedChemExpress. Palmitate (PA, purity > 98%, SP8060) was obtained from Solarbio Science & Technology. ERK antibody (AF0155), p-ERK antibody (AF1015), JNK antibody (AF6318), p-JNK antibody (AF3318), p38 antibody (AF6456), p-p38 antibody (AF4001) and insulin antibody (AF5109) were purchased from Affinity (Shanghai, China). Bcl-2 antibody (A0208), Bax antibody (A19684) and cleaved caspase 3/caspase 3 antibody (A19654) were obtained from Abclonal (Wuhan, China). β-actin antibody (60008-1-Ig), goat anti rabbit IgG (SA00001-2), and goat anti mouse IgG (SA00001-1) were purchased from Proteintech. (Wuhan, China).

### Ethic statement

All animal experiments were approved by the Animal Ethics Committee of the Heilongjiang University of Chinese Medicine (Harbin, China). All operations were conducted according to the Guild to the care and use of experimental animals.

### Animals and treatment

Male Sprague–Dawley (SD) rats (8 week old) were obtained from Liaoning changsheng Biotechnology and housed with free access to water and food at the temperature of 25 ± 1 °C and the humidity of 44–55% on a 12:12 light–dark cycle. After one-week acclimation, rats were randomly divided into 3 groups (n = 6 for each performed analysis). Rats in control group were maintained on a regular diet (RD, 3.85 kcal/g, 20% kcal protein, 70% kcal carbohydrates and 10% kcal fat; MD12031, Medicience) for 16 weeks. Rats in IGT group and IGT + PAL group were fed with a high fat diet (HFD, 4.73 kcal/g, 20% kcal protein, 35% kcal carbohydrates, 45% kcal fat; MD12032, Medicience) for 16 weeks. An oral glucose tolerance test (OGTT) was performed for the determination of HFD-induced alterations in glucose tolerance after six-week treatment of HFD. At the 7th week, rats in IGT + PAL group received an oral administration of PAL (40 mg/kg/day, dissolved in 0.5% sodium carboxymethylcellulose) for the 10 consecutive weeks with remained HFD, while rats in control and IGT group received the same volume of normal saline. Body weight and food intake were measured every 2 weeks after PAL administration. The detection of fasting blood-glucose and OGTT were conducted at the end of the16th week.

### Detection of serum samples

For OGTT, rats were fasted for 12 h and followed by an oral administration of glucose load (2 g/kg of body weight). The blood glucose (BG) levels were determined by a blood glucose test strip (Sinocare, Changsha, China) in tail blood at 0, 30, 60 and 120 min after glucose treatment. The area under the curve (AUC) was calculated using the following equation: AUC = 0.5 × (BG0 + BG30)/2 + 0.5 × (BG30 + BG60)/2 + 1×(BG60 + BG120)/2 according to the previous report [20]. The SD rats were fasted overnight and sacrificed at the 16th week. The fast blood glucose level was measured by glucometer (Sinocare, Changsha, China). The levels of insulin in serum were determined by ELISA kit (CEA448Ra, USCN Life Science) according to the manufactures’ protocol. The HOMA-IR was calculated as follows: fasting blood glucose (mmol/L) × fasting insulin (mIU/L)/22.5.

### Immunohistochemical (IHC) staining

The pancreatic tissues were fixed in 10% formaldehyde solution at 4 °C overnight. Sections were obtained after the paraffin embedding and slicing. For IHC staining, the sections were dewaxed and boiled in sodium citrate solution at for 10 min to perform antigen retrieval. Subsequently, the sections were blocked with normal goat serum (SL038, Solarbio, Beijing, China) at room temperature for 15 min and incubated with anti-insulin (1:200) at 4 °C overnight. The negative serum was used as a control to confirm the specificity of insulin staining. The incubation of the HRP-conjugated goat anti-rabbit IgG (1:500, **#**31460, thermoFisher) was subsequently conducted at 37 °C for 1 h. The sections were then colored using DAB (DA1010, Solarbio Biotechnology) and co-stained with hematoxylin (H8070, Solarbio Biotechnology). The micrographs were acquired using microscope (Olympus DP73, Tokyo, Japan). The β cell mass was calculated as follows: total area of a section of pancreas that stains positive for insulin (beta cell) divided by the total pancreas section area (beta-cell area, %), and multiplied by the pancreas weight (mg) that was taken when the animal was sacrificed [21].

### INS-1 cell culture and treatments

Rat insulinoma beta cell INS-1 was obtained from Zhong Qiao Xin Zhou Biotechnology (Shanghai, China). Cells were cultured in RPIM-1640 (ZQ206, Zhong Qiao Xin Zhou Biotechnology) containing 10% FBS in a 5% CO_2_ humid incubator at 37 °C. INS-1 cells were incubated with different doses of PAL (0 μg/ml, 10 μg/ml and 20 μg/ml) for 48 h to determine the most effective dose of PAL. Cells were subsequently treated with indicated PA (0.5 mM) for 24 h to establish a cellular model of IGT followed with incubation of PAL (20 mg/ml)/ERK inhibitor (10 μM)/JNK inhibitor (10 μM) together with or without glucose stimulation.

### MTT assay

Cells were seeded in 96-well plate with the density of 5 × 10^3^/well. After the pretreatments, cells were removed from the suspension and treated with 0.5 mg/ml MTT for 4 h in the dark in a 5% CO_2_ humid incubator at 37 °C. Subsequently, the precipitates were dissolved in 150 μL dimethyl sulfoxide (DMSO, KGT5131, KeyGen Biotech, Nanjing, China). The absorbance was measured at 570 nm on a microplate reader (ELX-800, BIOTEK, USA).

### Insulin secretion assay in INS-1 cells

INS-1 cells were starved in glucose–free RPMI 1640 for 2 h after the pretreatments, and then washed twice in HEPES-balanced Krebs–Ringer bicarbonate (KRB) buffer containing 2 g/L bovine serum albumin ((KRB/BSA) before the exposure to 8.3 mM glucose (in KRB buffer) for 1 h [22]. The levels of insulin secretion were measured by ELISA according to the manufacture’s instruction (CEA448Ra, USCN Life Science, Wuhan, China).

### Western-blot analysis

Total protein was extracted from the cells by RIPA lysis buffer (Beyotime) containing 1% PMSF (Beyotime) on ice. The concentration of total protein was determined by BCA Protein Quantification Kit (P0009, Beyotime). Protein samples were loaded on sodium dodecyl sulfate–polyacrylamide gel electrophoresis (SDS-PAGE) and transferred to a polyvinylidene fluoride membrane (PVDF), the membrane was then blocked with 5% (M/V) dissolved skimmed milk powder for 1 h. Subsequently, the membrane was incubated with anti-ERK (1:1000), anti-p-ERK (1:1000), anti-JNK (1:1000), anti-p-JNK (1:1000), anti-p-p38 (1:1000), anti-p38 (1:1000), anti-Bcl-2 (1:1000), anti-Bax (1:1000), anti-cleaved caspase 3/caspase 3 (1:1000) and anti-β-actin (1:2000) antibodies at 4 °C overnight. The incubation of HRP-conjugated secondary antibody (1:10000) was next performed for 45 min at 37 °C after the washing with TBST. The bands were finally visualized with ECL reagent (E003, 7Sea biotech). The optical density of each band was analyzed with Gel-Pro-Analyzer 4. Data were normalized to β-actin.

### TUNEL and insulin immunofluorescent staining

Sections were prepared as previously described in IHC staining. Subsequently, the incubation of 0.1% Trition X-100 was performed for 8 min to get permeabilization. After the antigen retrieval, the staining with TUNEL reaction solution (Enzyme solution: Label solution = 1: 9) was conducted on sections for 60 min in the dark at 37 ℃. After rising in PBS for three times, sections were blocked with normal goat serum at room temperature for 30 min, and next incubated with primary antibody anti-insulin (1:200) at 4 ℃ overnight. After washing with PBS, the incubation with Cy3-conjugated secondary antibody (1:200, A0516, Beyotime) was performed for 60 min followed by the staining of DAPI. The images were capture by the fluorescence microscope (OLUMPUS, DP73, Japan).

### Flow cytometry

The cell apoptosis was analyzed using the Apoptosis Detection Kit (C1062, Beyotime Biotechnology) by flow cytometry. Cells were removed from the suspension after centrifugation at 1000 g for 5 min. cells were then resuspended with 195 μl Annexin V-FITC binding buffer. On the following step, 5 μl Annexin V-FITC and 10 μl PropidiumIodide were added and cells were incubated with the mixture for 15 min on ice in the dark. The apoptotic rate was analyzed by the flow cytometry (NoyoCyte, USA).

### Statistical analysis

The data were represented as mean ± SD and analyzed using GraphPad Prism 7.0. One-way ANOVA following by the Tukey’s tests was used to analyze the differences among multiple groups. p < 0.05 was considered to represent statistically significance.

## Results

### Palmatine alleviates the impaired glucose tolerance (IGT) induced by high fat diet

**Fig. 1:**
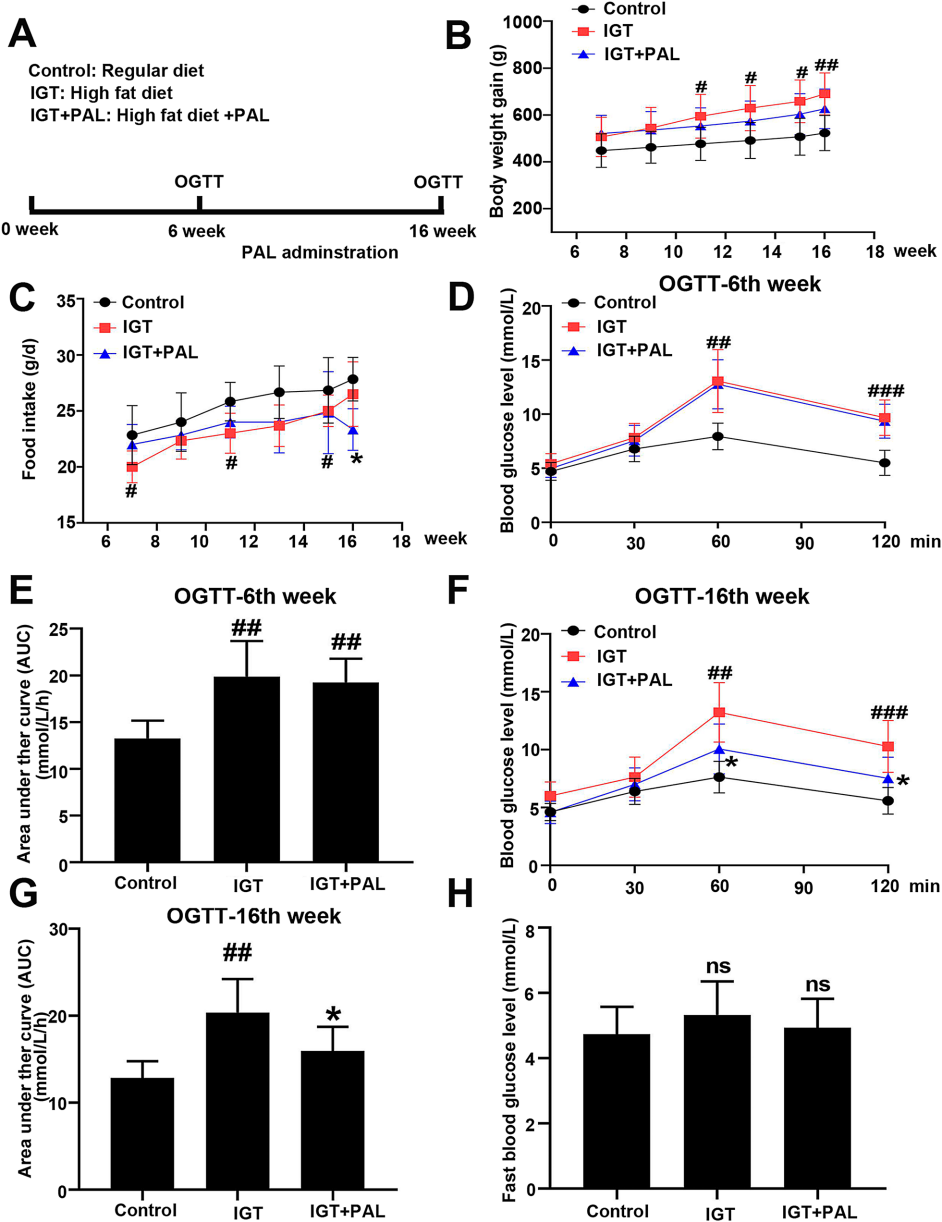
Effect of PAL on impaired glucose tolerance in high fat diet induced rats. **a** Animal experiment design. **b** Changes in body weight of three groups. **C** Changes in food intake of three groups. **d** Blood glucose response to a gastric glucose load at the 6th week. **e** Area under the curve of blood glucose at the 6th week. **f** Blood glucose response to a gastric glucose load at the 16th week. **g** Area under the curve of blood glucose at the 16th week. **h** Changes in fast blood glucose of three groups. Values are expressed as mean ± SD. n = 6. Compared with control group: ^#^P < 0.05, ^##^P < 0.01, ^###^ P < 0.001, ^####^ P < 0.0001; Compared with IGT group, *P < 0.05, **P < 0.01, *** P < 0.001, **** P < 0.0001

High fat diet (HFD) was provided to induce impaired glucose tolerance in SD rats (Fig. 1a). The body weight and food intake were measured every 2 week after the administration of PAL. As shown in Fig. 1b, HFD rats displayed a significant increase in the body weight compared to the control, while rats fed with HFD disclosed lower food intakes than that in RD rats (Fig. 1c). After 6 weeks of HFD access, OGTT was conducted to confirm the occurrence of glucose intolerance in HFD rats. As shown in Fig. 1d–e, significantly elevated 2-h blood glucose level and the area under the curve (AUC) was observed in HFD rats when compared to control (rats with regular diet), suggesting that high fat diet successfully mimic the IGT model in vivo. An oral administration of PAL was performed for the following 10 weeks, and another OGTT indicated the alleviation of IGT with significantly decreased 2-h blood glucose level and the area under the curve (AUC) (Fig. 1f–g). Moreover, no significant change in the fast blood glucose was found at the end of 16th week (Fig. 1h).

### Palmatine restricts the insulin resistance and β-cell mass loss in high fat diet induced rats

**Fig. 2:**
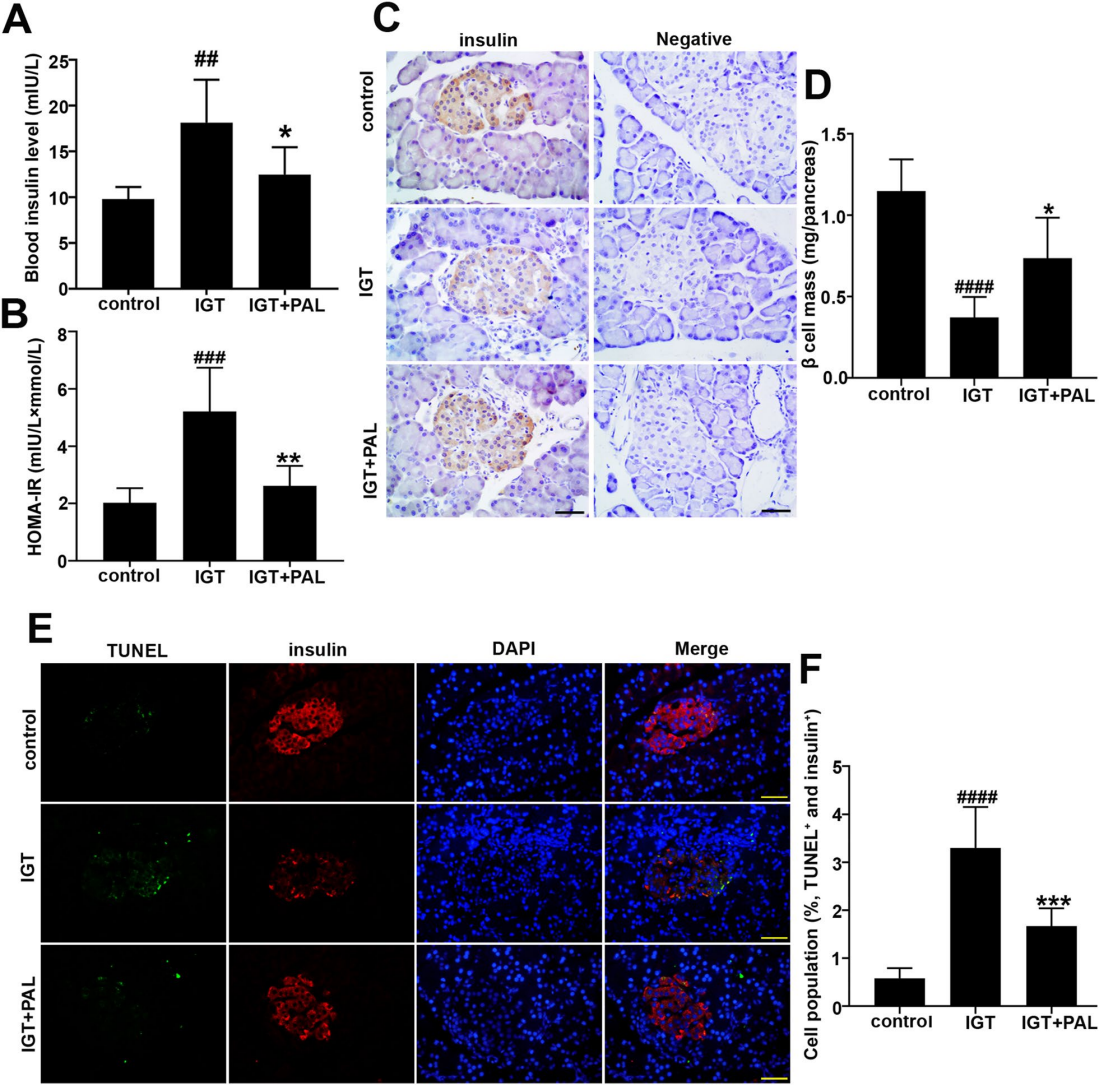
Effect of PAL on the insulin resistance and β-cell mass in high fat diet induced rats. **a**The blood insulin levels of three groups. **b** The HOMA-IR in three groups. **c** The β cells mass in each group determined by immunohistochemical staining. Scale bar: 50 μm. **d** Quantification of the β cell mass. **e** Cell apoptosis in β cells determined by TUNEL/insulin immunofluorescent staining. Scale bar: 50 μm. **f** Quantification of TUNEL-postive β cells. Values are expressed as mean ± SD. n = 6. Compared with control group: ^#^P < 0.05, ^##^P < 0.01, ^###^ P < 0.001, ^####^ P < 0.0001; Compared with IGT group, *P < 0.05, **P < 0.01, *** P < 0.001, **** P < 0.0001

As shown in Fig. 2a, the HFD induced rats displayed a significant increase in blood insulin level when compared with control group. On the contrary, administration of PAL showed significant inhibitory effect on this alteration. Further analysis of HOMA-IR suggested the protective role of PAL in the insulin resistance induced by HFD (Fig. 2b). Meanwhile, the β-cell mass in different groups were determined by immunohistochemical staining and relative quantification. Besides, the specificity of insulin staining was confirmed by the negative serum incubation. As shown in Fig. 2c–d, significant loss of islet β cell was observed in IGT group when compared with control, and PAL conversely restored the loss of β cell mass with predominantly increased area that stains positive for insulin. To investigate whether β cell apoptosis contributed to the β cell loss in IGT model, TUNEL/insulin immunofluorescent staining was performed. Accordingly, cell population of TUNEL-positive β cells was significantly increased in IGT model, while PAL treatment remarkably reduced the apoptotic rates on the contrary (Fig. 2e–f).

### Palmatine suppresses the MAPK signaling in high fat diet-induced rats

**Fig. 3:**
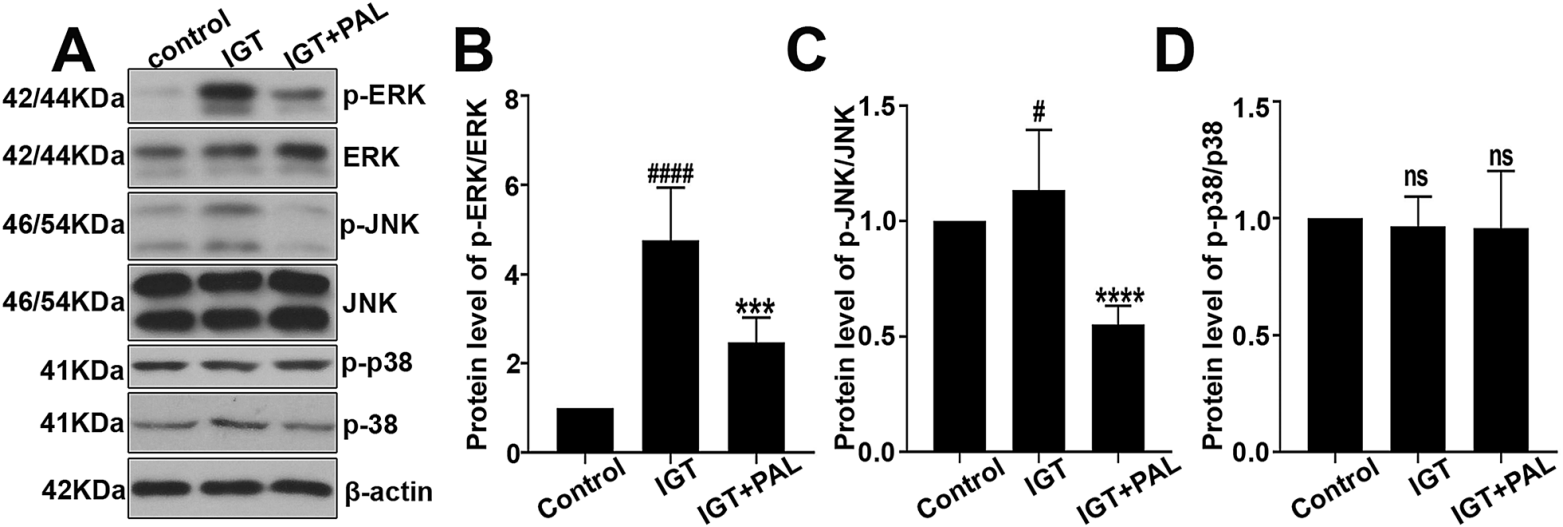
Effect of PAL on the MAPK signaling in high fat diet-induced rats. **a** The expression levels of p-ERK (Thr202/Tyr204), ERK, p-JNK (Thr183/Tyr185), JNK, p-p38 (Thr180/Tyr182) determined by western-blot. **b** The quantitative analysis of p-ERK/total ERK expression level. **c** The quantitative analysis of p-JNK/total JNK expression level. **d** The quantitative analysis of p-p38/total p38 expression level. Values are expressed as mean ± SD. n = 6. Compared with control group: ^#^P < 0.05, ^##^P < 0.01, ^###^ P < 0.001, ^####^ P < 0.0001; Compared with IGT group, *P < 0.05, **P < 0.01, *** P < 0.001, **** P < 0.0001

Three important molecules about MAPK signaling including the ERK, JNK and p38 were investigated in our study. Particularly, the protein levels of p-ERK, ERK, p-JNK, JNK, p-p38 and p38 were measured by western-blot analysis along with quantification (Fig. 3a–d). We found that the phosphorylation of ERK and JNK was promoted in IGT model, and PAL treatment significantly reduced the protein levels of p-ERK and p-JNK in the contrast. However, no significant alterations were observed in the expression level of p-p38 and p38.

### Palmatine inhibits the MAPK signaling in palmitate (PA) treated INS-1 cells

**Fig. 4:**
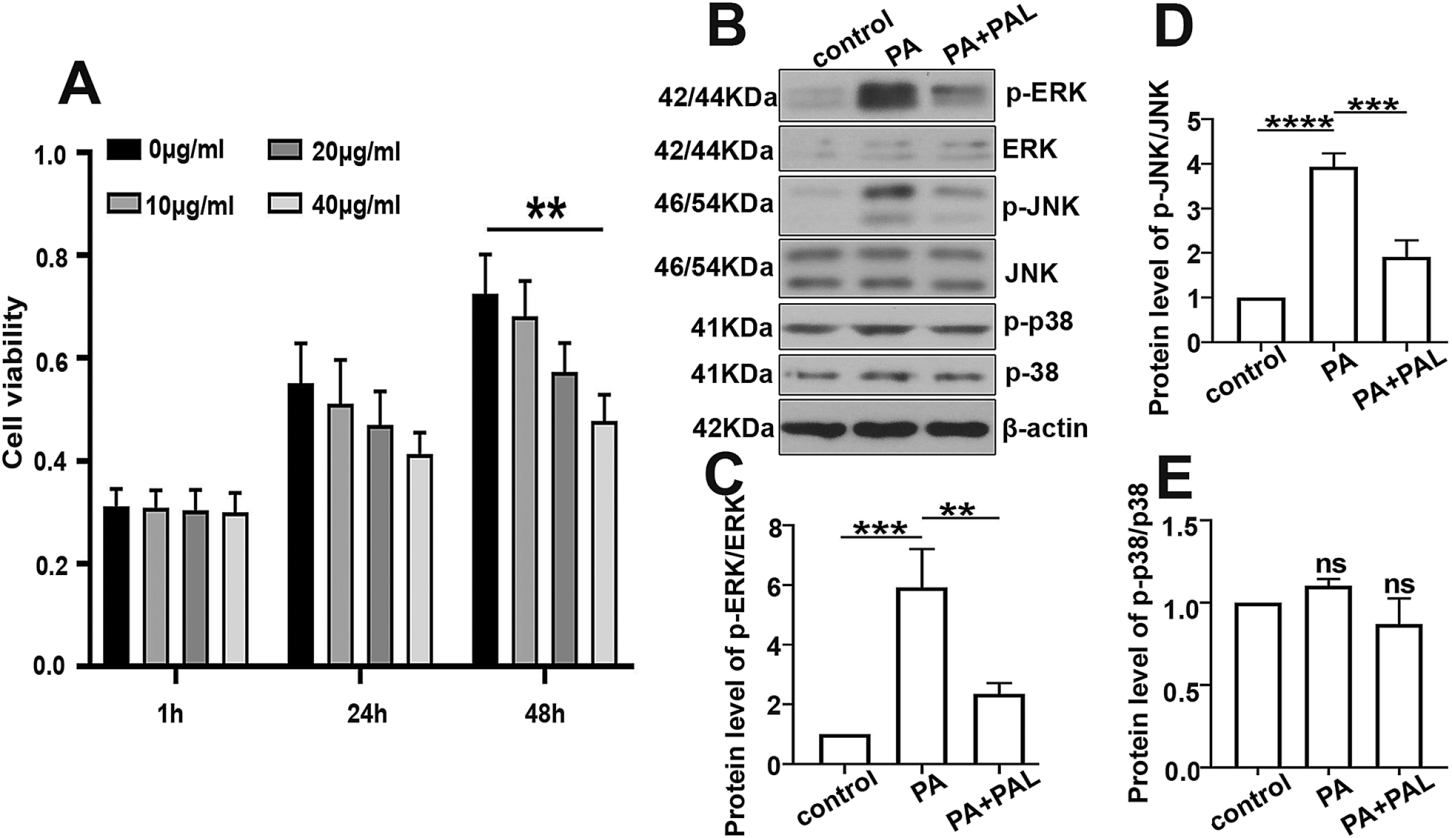
Effect of PAL on the MAPK signaling in palmitate (PA) treated INS-1 cells. **a** Cells were incubated with different doses of PAL (0 μg/ml, 10 μg/ml, 20 μg/ml, 40 μg/ml) for 1 h, 24 h and 48 h. Cell vitality was determined by MTT assay. **b** The expression levels of p-ERK (Thr202/Tyr204), ERK, p-JNK (Thr183/Tyr185), JNK, p-p38 (Thr180/Tyr182) determined by western-blot along with the quantification analysis of p-ERK/total ERK (**c**), p-JNK/total JNK (**d**) and p-p38/total p38 (**e**). Values are expressed as mean ± SD. n = 3.*P < 0.05, **P < 0.01, *** P < 0.001, **** P < 0.0001

MTT assay was performed to indentify the appropriate concentration and incubation time for PAL on INS-1 cells, and we found no significant difference in cell viability after the incubation of PAL (20 μg/ml) for 48 h. Subsequently, a cellular model of glucose intolerance was built up by palmitate incubation (PA, 0.5 mM) for 24 h followed by the PAL intervention. It was shown that the protein levels of p-ERK, p-JNK was significantly increased after the stimulation of PA, while PAL treatment conversely reduced their expressions in PA induced INS-1 cells (Fig. 4b–d). However, no significant alterations were observed in the expression level of p-p38 and p38 (Fig. 4e).

### Palmatine promotes the secretion of insulin in INS-1 cells

**Fig. 5:**
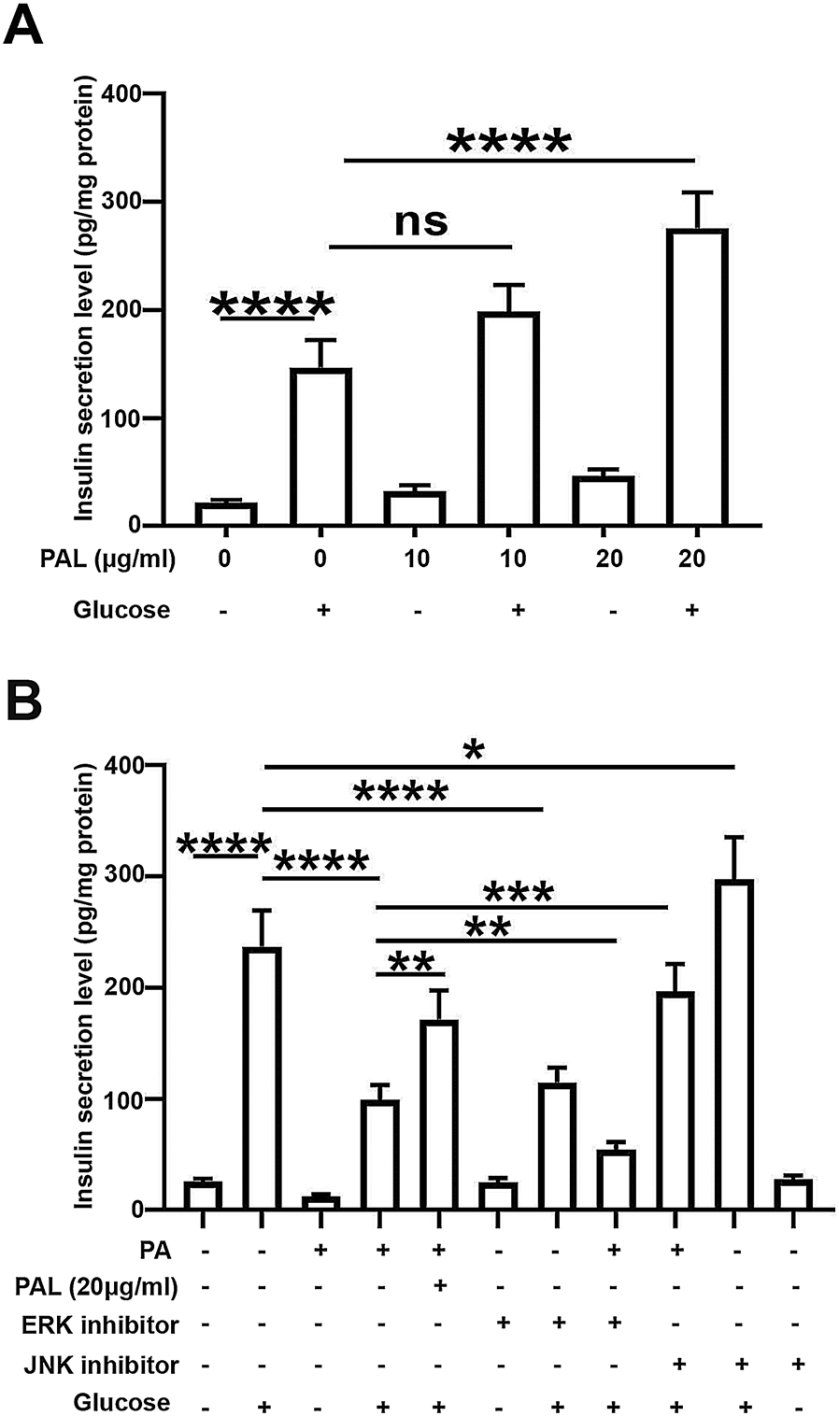
Effect of PAL on the insulin secretion in INS-1 cells (**a**) INS-1 cells were incubated with different doses of PAL (0 μg/ml, 10 μg/ml, 20 μg/ml) for 48 h. The insulin secretion was determined upon the glucose stimulation, and then normalized to the protein concentration. **b** PA- induced cells were respectively incubated with PAL (20 μg/ml), ERK inhibitor PD98059 (10 μM), and JNK inhibitor SP600125 (10 μM). The insulin secretion was subsequently assessed upon glucose stimulation, and then normalized to the protein concentration. Values are expressed as mean ± SD. n = 3.*P < 0.05, **P < 0.01, *** P < 0.001, **** P < 0.0001

INS-1 cells were treated with increasing concentrations (0 μg/ml, 10 μg/ml, 20 μg/ml) of PAL for 48 h followed by the 1-h challenge of 8.3 mM glucose to detect the insulin secretion level. Our ELISA analysis indicated that PAL (20 μg/ml) significantly enhanced the secretion of insulin when compared to control (Fig. 5a). Besides, PA-treated INS-1 cells showed much lower insulin secretion level than that in control after the glucose stimulation. Meanwhile, we found that PAL treatment significantly promoted the release of insulin in PA-induced cells (Fig. 5b). Previous study has indicated that the deficiency of ERK displays protective effect on insulin resistance and obesity [23], and the JNK signaling is also involved in the secretion of insulin [24]. Thus ERK inhibitor (PD98059, 10 μM) or JNK inhibitor (SP600125, 10 μM) was employed to determine whether PAL facilitated the insulin release in an ERK/JNK dependent manner. As shown in Fig. 5b, JNK inhibitor displayed the same stimulative effect on insulin secretion as PAL, while ERK inhibitor significantly reduced the level of insulin secretion on the contrary, which indicated that PAL enhance the insulin secretion by inhibiting the JNK pathway.

### Palmatine restrains the β cell apoptosis in palmitate (PA) treated INS-1 cells

**Fig. 6:**
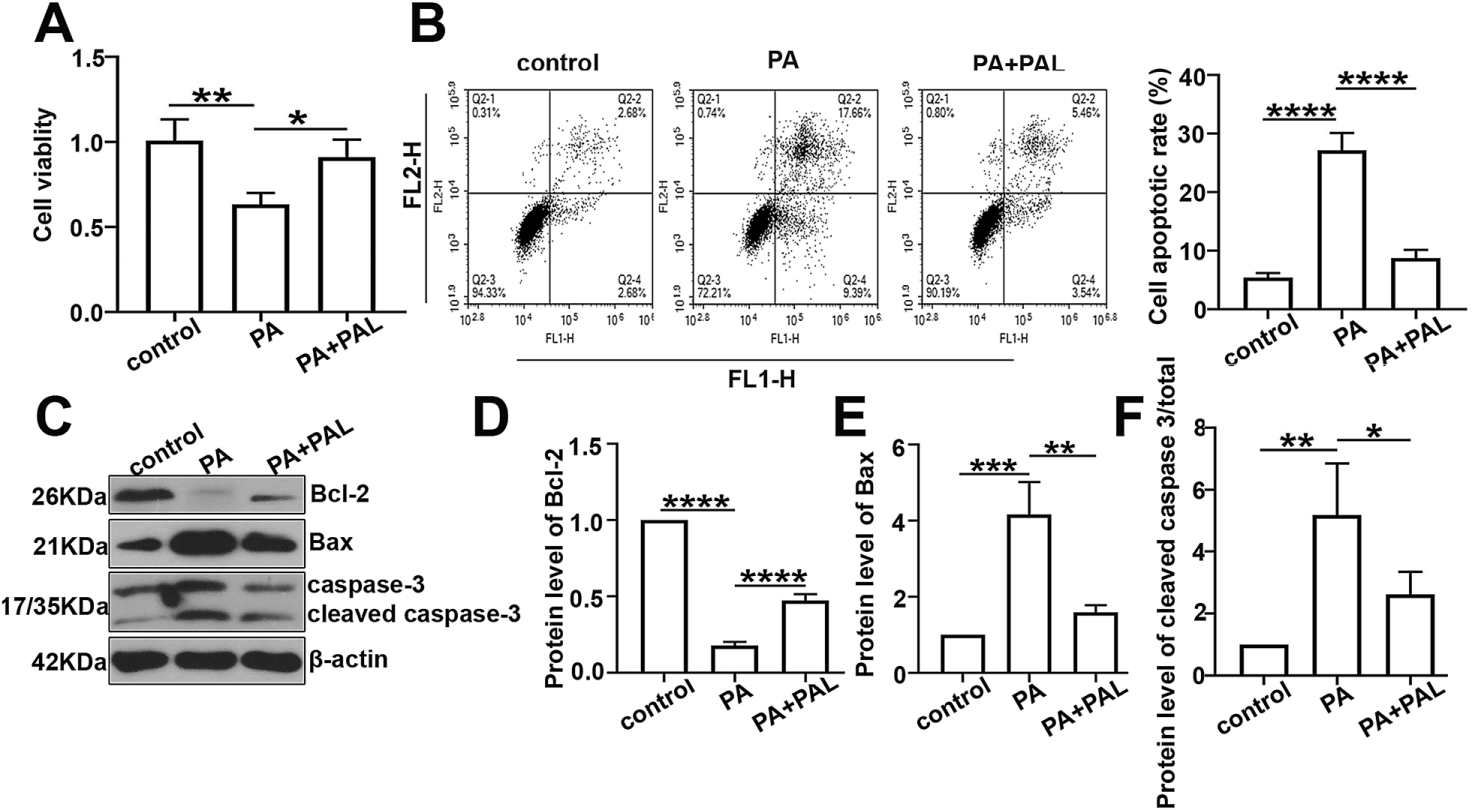
Effect of PAL on cell apoptosis in palmitate (PA) treated INS-1 cells. **a** The cell viability in each group determined by MTT assay. **b** The cell apoptotic rate assessed by flow cytometry along with quantification. **c** The protein levels of Bcl-2, Bax, cleaved caspase-3 and caspase-3 measured by western-blot analysis. **d** Quantification analysis of Bcl-2 expression level. **e** Quantification analysis of Bax expression level. **f** Quantification analysis of cleaved caspase-3/total caspase-3 expression level. Values are expressed as mean ± SD. n = 3.*P < 0.05, **P < 0.01, *** P < 0.001, **** P < 0.0001

Cell viability was significantly restricted in PA treated cells while reversed after PAL intervention according to the MTT assay (Fig. 6a). The cell apoptosis in PA treated cells was investigated by the flow cytometry. As shown in Fig. 6b, the apoptotic rate of PA induced cells was significantly elevated, while PAL treatment conversely reduced the cell population with apoptosis. Furthermore, the protein levels of Bcl-2, Bax, cleaved caspase-3 and caspase-3 were respectively analyzed by western-blot with quantification (Figure c–f). PA treated INS-1 cells indicated up-regulated expressions of Bax and cleaved caspase-3, while PA displayed an inhibitory effect on the expression of Bcl-2. Moreover, treatment with PAL significantly compensated these alterations, suggesting that PAL could significantly restrain the β cell apoptosis in PA treated INS-1 cells.

## Discussion

Impaired glucose tolerance (IGT) is a progressive metabolic disease which is characterized with above normal plasma glucose responses to an oral glucose load [25]. This kind of abnormality not only accelerates the proceeding of diabetes but also predicts it. In our study, high fat diet (HFD) was adopted to establish a model for impaired glucose tolerance in SD rats. Oral administration of PAL was further performed to indentify the effect of PAL on glucose intolerance. Given the results obtained in our study, we demonstrated that PAL alleviated the impaired glucose tolerance in high fat diet induced rats. High fat diet has been a universally accepted method to establish IGT model which was originally introduced by Surwit et al. in 1988 [26]. The model has been used in studies on developing new treatments of IGT and T2D. Remarkable augment in body weight was found in HFD induced rats, indicating the potential correlation between IGT and obesity [27]. Actually, the prevalence of IGT is much higher among the children and adolescents with obesity, which suggests that IGT is a kind of obesity-related metabolic abnormality [28]. Additionally, OGTT was performed in our study and indicated a significant relief of glucose intolerance after PAL treatment on the basis of the plasma glucose level before and 2 h after an oral glucose load [29]. Interestingly, we also found that the fast blood glucose was barely altered in rats with HFD diet at the 16th week, and we thought it is because of the restricted hypoglycemic effect of insulin during the early event of IGT.

It is well established that the dysfunction of islet β cell will bring about the onset of diabetes and prediabetes [30]. Accordingly, we here demonstrated that HFD induced rats displayed higher insulin resistance and defective β cell mass, while administration of PAL showed a compensatory effect on these alterations in our study. Interestingly, the blood insulin level at the 16th week was determined to be significantly increased in HFD rats and reduced after PAL treatment. Actually, rats under the status of IR will have a compensatory secretion of insulin, also named as hyperinsulinemia, to achieve the balance of glucose [31, 32]. Moreover, the elevated HOMA-IR is widely accepted as the hallmark of insulin resistance, which is defined as an abnormal condition that insulin exerts a much lower biological effect than expected, and it is resulted from the defects of multiple biological processes including insulin-stimulated glucose, glycogen synthesis and glucose oxidation [33]. Additionally, the β-cell secretive capacity plays an important role in the regulation of blood glucose homeostasis. It is noteworthy that insulin release will elevate to compensate insulin resistance so that to maintain euglycemia, and subsequently falls down to elevate the plasma glucose levels [34], making it a very complicated physiological process in organism. However, maintenance of functional β cells has been accepted as a key point in insulin secretion. Several studies have implied that the insufficient insulin secretion is partly due to the decreased β cell mass [35], and suggested that apoptotic β cell death is the major contributor of the cell mass loss [36]. Basically, the relieved β cell apoptosis was determined in our study after PAL treatment, further validating the critical role of apoptosis in healthy β cell mass.

PAL is a typical isoquinoline alkaloid with various pharmacological functions. It has shown the protective effect on Alzheimer’s disease [37], and exerts inhibitory effect on inflammatory reaction, tumorigenesis and oxidative response [9, 17, 38]. Interestingly, our study demonstrated that PAL could regulate the MAPK signaling by inhibiting the ERK and JNK pathways based on the experiment in vivo and vitro. Accordingly, the MAPK cascade mainly consists of ERK, JNK and p38 signaling, and these MAPK members are usually activated through their phosphorylation. MAPK signaling has been reported to participate in numerous physiological processes such as the cell proliferation, differentiation, apoptosis and even insulin action [39, 40]. It should be noted that the selectively blocking of ERK signaling is documented to be beneficial for the alleviation of insulin resistance in T2D [23], and the absence of JNK has been determined to improve the insulin sensitivity [41]. Meanwhile, insulin secretion assay in our study convinced that PAL could promote the insulin secretion in INS-1 cells, and we consequently investigated whether PAL promoted the insulin release by inactivating ERK or JNK signaling using the inhibitor of ERK or JNK (PD98059 or SP600125). We finally figured out that the PAL-mediated promotion of insulin secretion was dependent on the restriction of JNK cascade rather than ERK signaling. Actually, ERK signaling restraining has been reported to negatively regulate the insulin secretion, while the deficiency of JNK is determined to facilitate it [42, 43], which is consistent with our findings.

Importantly, the defective β cell function is believed to result in the reduction of insulin release from islet β cells. We thus prefer that the inhibited apoptotic β cell death in PAL treated cells is another contributor to the promotion of insulin release from INS-1 cells. Generally, PAL treatment showed a significant inhibitory effect on the cell population with apoptosis in palmitate (PA) induced cellular model of IGT [44]. Besides, alterations in the expressions of pro-apoptotic or anti-apoptotic proteins such as Bax and Bcl-2 suggested that PAL could alleviate the PA-induced apoptotic β cell death. Actually, exposure of islet β cells to PA has been confirmed to impair insulin release and even leads to the occurrence of β cell apoptosis [45]. In addition, MAPK signaling also has a critical role in the regulation of apoptosis [39]. For instance, Hydrogen sulfide could reduce the apoptosis in LPS induced diaphragm dysfunction by inhibiting the MAPK signaling with significantly down-regulated expression of p-ERK, p-JNK and p-p38 [46]. Specially, ERK activity is strongly associated with multiple anti-proliferative events like apoptosis, and ERK kinase inhibitor has been demonstrated to alleviate the ischemia/reperfusion induced cell apoptosis in myocardium [47], suggesting that PAL–mediated relief of β cell apoptosis might be dependent with the restriction of ERK signaling, and subsequently associated with PAL mediated promotion of insulin release. More importantly, apoptosis serves as a process of cellular self-destruction and plays an important apart in the balance between cell proliferation and death, while Inflammation is documented to be an immune system induced response to the infection or tissue damage. The inflammatory response has been accepted as an essential contributor to the host defense and tissue repair [48]. In general, apoptosis is primarily modulated by the apoptotic genes, while it is also influenced by some other molecules such as the inflammatory mediators. In general, tumor necrosis factor-α (TNF-α) has been reported to induce pancreatic apoptosis in acute pancreatitis, and the transcription factor (NF-κB) is also proved to participate in the regulation of apoptosis [49]. It is validated that NF-κB inhibitor PDTC could attenuate the LPS-induced cell apoptosis in MAC-T cells [50]. Accordingly, PAL possesses strong anti-inflammatory properties in many infection related disease. Thus, it is of great potential that PAL might alleviate the β cell apoptosis in IGT model by inhibiting the inflammatory response.

## Conclusion

In summary, our findings reveal the protective role of PAL in high fat diet induced impaired glucose tolerance with reduced insulin resistance and promoted islet β cell mass in vivo. Additionally, PAL significantly restricted the MAPK signaling via ERK and JNK cascades in IGT models of vivo and vitro. Furthermore, we found that PAL could promote the insulin secretion in INS-1 cells by inhibiting the JNK signaling and apoptotic β cell death in INS-1 cells.

## Data Availability

All data generated or analyzed during this study are included in this article.

## Abbreviations

IGT: Impaired glucose tolerance
PAL: Palmatine
PA: Palmitate
HFD: High fat diet
OGTT: Oral glucose tolerance test
IR: Insulin resistance
HOMA-IR: Homeostatic model assessment of insulin resistance
T2D: Type 2 diabetes
TCMs: Traditional Chinese medicines
BG: Blood glucose
